# Chest Radiotherapy Associated Cardiotoxicity: An Update

**DOI:** 10.1007/s11886-026-02437-6

**Published:** 2026-10-27

**Authors:** Raksheeth Agarwal, Antony Gonzalo Gonzales-Uribe, Lili Zhang

**Affiliations:** 1 Cardiology Division, Hartford Hospital, University of Connecticut, Hartford, CT 06102 USA; 2 Department of Medicine, Jacobi Medical Center, Albert Einstein College of Medicine, 1400 Pelham Parkway South, Bronx, NY 10461 USA; 3 Cardiology Division, Department of Medicine, Montefiore Medical Center/Albert Einstein College of Medicine, 111 E 210th St, Bronx, NY 10467 USA

**Keywords:** Cardio-Oncology, Radiation-Induced Heart Disease, Chest Radiotherapy

## Abstract

**Purpose of Review:**

Chest radiotherapy has improved cancer survival in various thoracic malignancies, including breast cancer, Hodgkin lymphoma, and lung cancer. However, inadvertent radiation exposure to the heart is associated with a broad spectrum of cardiovascular toxicities, collectively termed radiation-induced heart disease (RIHD). We aim to review the pathophysiology of RIHD and the current evidence for their incidence, risk factors, clinical management, and prevention strategies.

**Recent Findings:**

RIHD is mediated by oxidative stress, DNA damage, endothelial dysfunction, inflammation, and progressive fibrosis, leading to accelerated atherosclerosis, myocardial stiffening, valvular degeneration, pericardial disease, and arrhythmias. The risk, timing, and severity of cardiovascular complications are influenced by radiation dose and distribution, age at exposure, baseline cardiovascular risk, and concomitant cardiotoxic therapies. While younger patients may experience prolonged latency before clinical disease onset, older individuals and those with pre-existing cardiovascular disease often develop complications earlier, reflecting radiation-mediated acceleration of underlying pathology.

**Summary:**

This review summarizes the epidemiology, latency, clinical presentation, surveillance strategies, and management of the different forms of RIHD. It also discusses contemporary approaches to risk mitigation, including advances in radiation delivery techniques, aggressive cardiovascular risk factor modification, lifestyle interventions, and emerging pharmacologic strategies.

**Graphical Abstract:**

**Figure:**
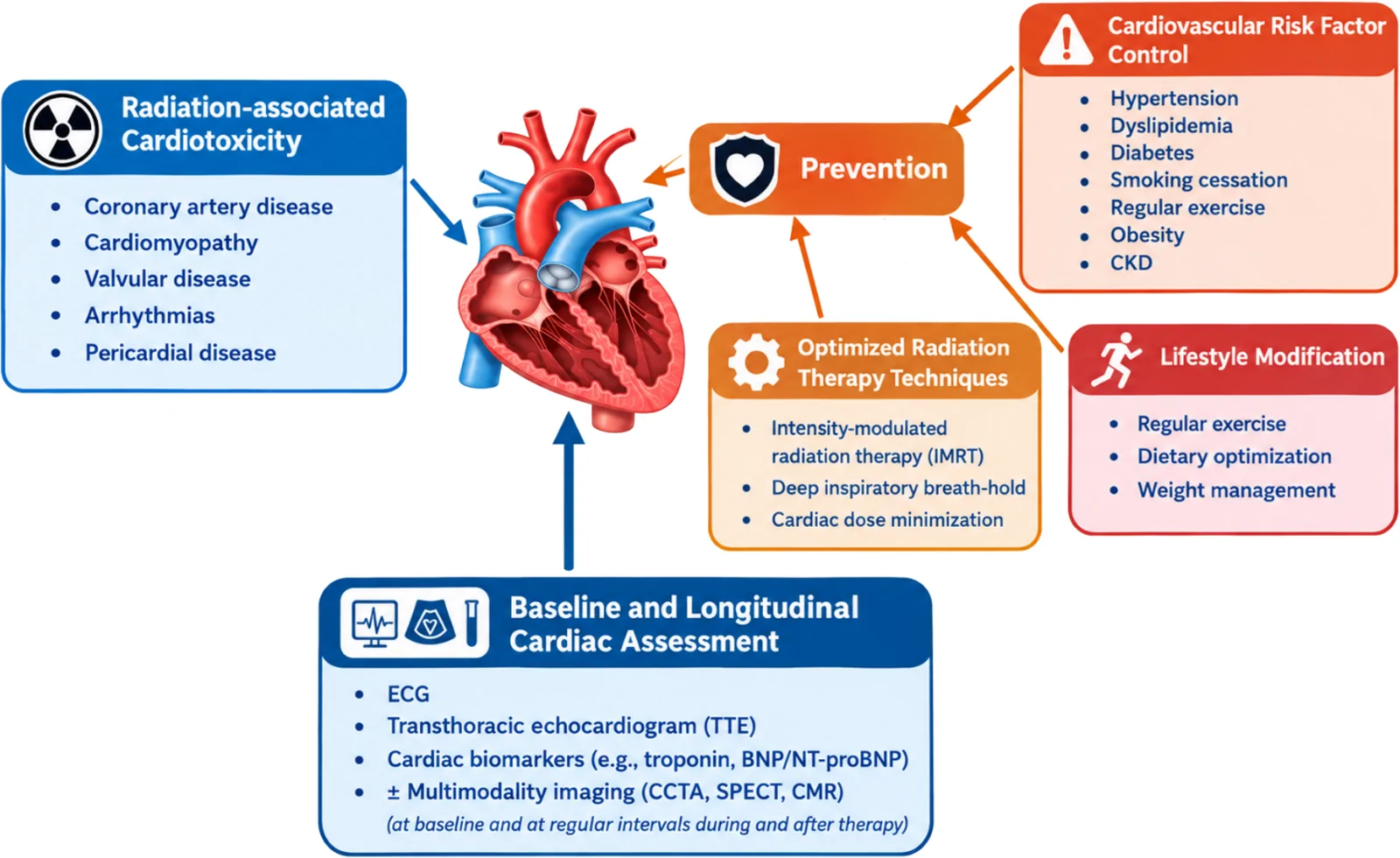

## Introduction

Advancements in the use of chest radiotherapy have improved survival in several thoracic malignancies including breast cancer, Hodgkin’s lymphoma, and lung cancer. However, the benefits of chest radiotherapy are limited by the development of adverse effects and toxicities in a large subset of patients. The recognition of heart disease occurring after irradiation of the mediastinum dates to the 1960s, primarily in the context of increasing survivors of Hodgkin’s lymphoma. Improvements in modern radiation therapy techniques have significantly reduced the incidence of radiation-induced heart disease (RIHD), yet it remains a critical concern for long-term cancer survivors and cancer patients with elevated risk of cardiovascular disease. Inadvertent irradiation of the heart and related cardiovascular structures can potentially lead to a broad spectrum of cardiotoxicities including coronary artery disease (CAD), valvular heart disease (VHD), cardiomyopathy, pericardial disease, and arrhythmias. We review the pathophysiological mechanisms involved in chest radiotherapy associated cardiotoxicity and the current evidence for their incidence, risk factors, clinical management, and current strategies to mitigate the risk.

## Pathophysiological Mechanisms

**Fig. 1:**
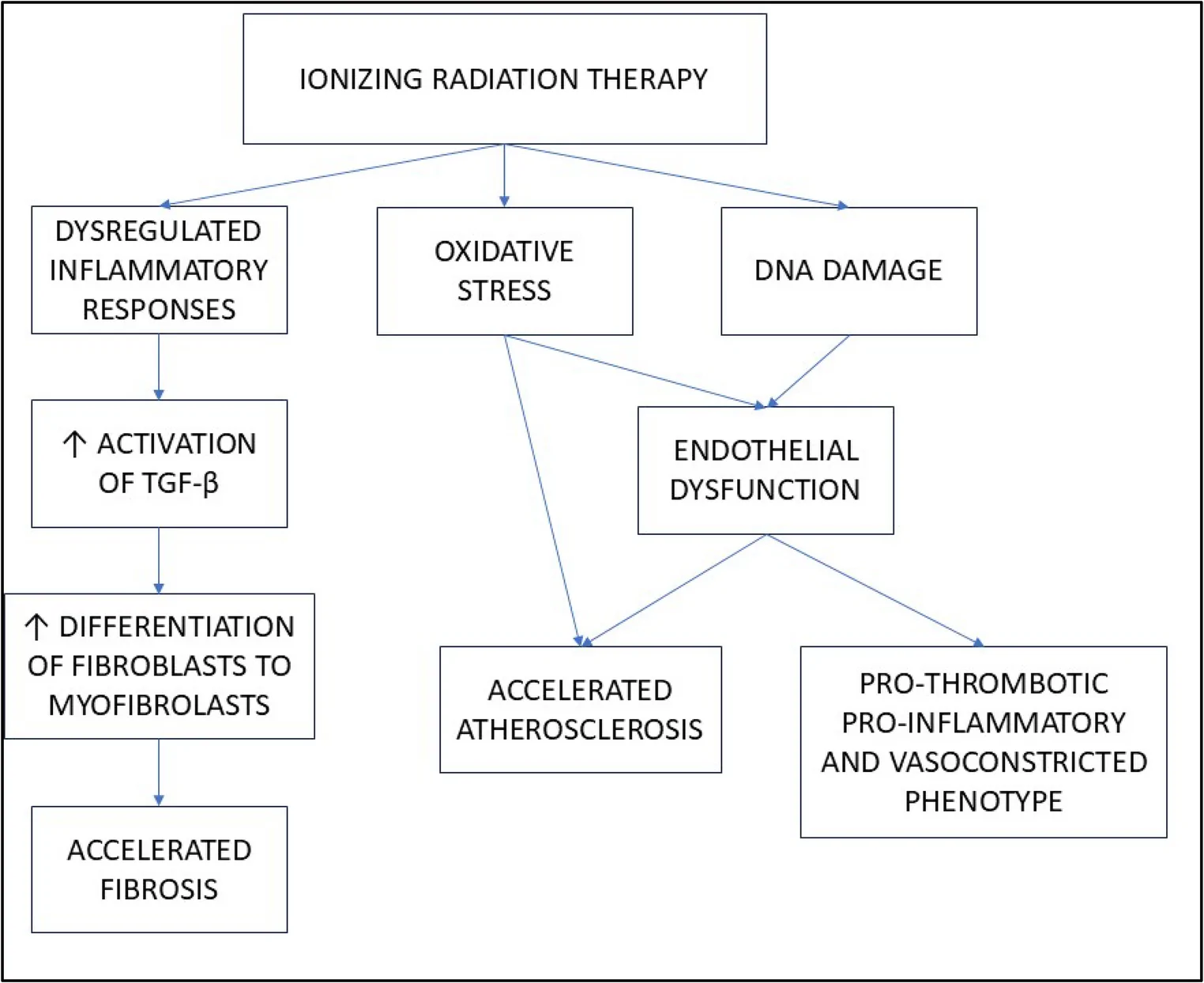
Pathophysiological mechanisms of ionizing radiation associated toxicity. DNA = Deoxyribonucleic Acid. TGF- ß = Transforming Growth Factor-Beta

Radiation therapy mediates cardiotoxicity by oxidative stress, DNA damage, endothelial dysfunction, and inflammation (Fig. 1). Radiation leads to rapid hydrolysis of water molecules resulting in the formation of free radicals and reactive oxygen species (ROS) [1]. Ionizing radiation causes deoxyribonucleic acid (DNA) alterations both directly and indirectly by oxidative stress [1, 2]. Radiation induced DNA damage mediates endothelial cell apoptosis via activation of genes like Ataxia Telangiectasia-Mutated (ATM) and p53 [1]. Oxidative stress also enhances endothelial dysfunction, which in turn results in a pro-thrombotic, vasoconstricted, and pro-inflammatory phenotype. Oxidative stress results in lipid peroxidation and formation of foam cells, and together with endothelial dysfunction, this ultimately leads to accelerated atherosclerosis and CAD [2]. 

An important hallmark chronic consequence of radiation toxicity is accelerated fibrosis. Dysregulated inflammatory cytokine signals and oxidative stress from ionizing radiation increase activation of transforming growth factor-beta (TGF-ß) [3]. TGF-ß promotes the differentiation of fibroblasts to myofibroblasts, which lead to excessive extracellular matrix depositions and collagen synthesis [2, 3]. This results in fibrotic changes in cardiac valves, the myocardium (causing reduced compliance and diastolic dysfunction), the pericardium, and the conduction system (resulting in arrhythmias).

## Cardiovascular complications

Chest radiotherapy associated cardiotoxicity can present in various domains, including coronary artery disease, valvular heart disease, cardiomyopathy, pericardial disease, and arrhythmias. Table 1 below summarizes the current knowledge on epidemiology, risk factors, monitoring, and management for each domain of cardiotoxicity, followed by text describing the current evidence for this information.

**Table 1:** Epidemiology, risk factors, monitoring, and management for radiotherapy associated cardiotoxicity

Cardiotoxicity domain	Epidemiology	Risk factors	Monitoring / management
Coronary Artery Disease	• Most common RT complication • Increased CAD, MI, ACS risk • Latency often 10–20 + years	• Higher cardiac dose • Left-sided breast RT • HTN, DM, dyslipidemia, smoking • Pre-existing CAD	• Screening every 5 years • Stress testing, CCTA, CAC • Aggressive risk factor control • Standard CAD therapy
Valvular Heart Disease	• Prevalence 2–37% • Aortic and mitral valves most affected • Incidence rises with time	• Higher valve/heart radiation dose • Anthracyclines • Left-sided RT	• TTE every 5 years, or more frequently in higher risk populations • Catheter-based interventions preferred
Cardiomyopathy	• Diastolic dysfunction (14–29%) most common • More direct relationship with HFpEF than HFrEF	• Higher radiation dose • Anthracyclines • CV risk factors • CAD	• Baseline ECG/TTE • Periodic TTE/CMR surveillance • Guideline-directed HF therapy
Pericardial Disease	• Effusions, chronic pericarditis, constriction • May occur years after RT	• Higher radiation dose • Larger radiation fields • Chemotherapy exposure	• TTE every 5 years • CT/CMR if constriction suspected • Standard pericardial interventions
Arrhythmias	• AF most common • Incidence ~ 5–21% depending on population	• Higher atrial radiation dose • Older age • Structural heart disease	• ECG at surveillance visits • Standard arrhythmia management • Enhanced monitoring for CIEDs during RT

*RT* Radiotherapy, *CAD* Coronary Artery Disease, *MI* Myocardial Infarction, *ACS* Acute Coronary Syndrome, *HTN* Hypertension, *DM* Diabetes Mellitus, *CCTA* Coronary Computed Tomography Angiography, *CAC* Coronary Artery Calcium, *TTE* Transthoracic Echocardiography, *HFpEF* Heart Failure with Preserved Ejection Fraction, *HFrEF* Heart Failure with Reduced Ejection Fraction, *CV* Cardiovascular, *ECG* Electrocardiogram, *CMR* Cardiac Magnetic Resonance Imaging, *AF* Atrial Fibrillation, *CIED* Cardiac Implantable Electronic Device

### Atherosclerosis and Coronary Artery Disease

#### Epidemiology

CAD is one of the most common cardiac complications of chest radiation therapy. In a matched case–control study by van Rosendael et al., 78 lymphoma survivors treated with mediastinal radiotherapy (mean radiation dose ~ 36 Gy; mean age 45 ± 11 years; median 16 years from exposure) underwent coronary computed tomographic angiography (CCTA) and were compared with 78 non-irradiated controls. CAD, defined as ≥ 50% coronary stenosis, was detected in 59% of irradiated patients versus 36% of matched controls, corresponding to an approximately 2.7-fold higher burden of multivessel disease in the irradiated group [4]. In another cohort of Hodgkin’s Lymphoma patients receiving chest radiation therapy, the incidence of significant CAD after 9 years was 10.4%. In lung cancer patients receiving thoracic radiotherapy (*n* = 35; mean age 57), the incidence of coronary artery calcium (CAC) was 48.6% compared with 24.6% in 65 matched non-irradiated controls (*P* = 0.01), with radiation exposure independently predicting new CAC development (adjusted OR 3.1; 95% CI 1.09–9.2) [5]. The rates of acute coronary syndromes are also elevated following thoracic radiation therapy. In a pooled analysis of prospective multicenter trials including patients with locally advanced non–small-cell lung cancer (NSCLC) treated with definitive radiation therapy, the cumulative incidence of acute coronary syndrome was approximately 4% at a median follow-up of 23 months, a rate higher than expected for age- and comorbidity-matched populations without thoracic irradiation [6]. Similarly, in a population-based cohort of lymphoma survivors, Salz et al. estimated a 10-year cumulative incidence of myocardial infarction (MI) of 3.9% among patients receiving chest radiation therapy, which increased to 10.2% in those with concurrent cardiovascular risk factors. In comparison, lymphoma survivors not exposed to radiation therapy had substantially lower MI risk, and radiation exposure remained independently associated with MI after adjustment for traditional cardiovascular risk factors [7]. 

#### Risk Factors

Several factors may enhance the risk of CAD in patients who have received chest radiation therapy, including cancer-related factors and traditional cardiovascular risk factors.

There is strong evidence for a dose-dependent relationship between chest radiation therapy and risk of CAD. A recent systematic review on childhood cancer survivors found that every additional 10 Gy units of radiation exposure doubles the risk of CAD 30 years after the radiation exposure [8]. Moreover, The Childhood Cancer Survivor Study found that exposure to cardiac radiation between 15 and 35 Gy increased the risk of MI by 2.4 times, while exposure above 35 Gy raised the risk by 3.6 times [9]. Nimwegen et al. also found a dose-dependent relationship between radiation exposure and CAD in Hodgkin Lymphoma survivors, with a 7.4% increased risk with every Gy of radiation [10]. This corroborates the landmark study by Darby, et al., which found a 7.4% increased risk of major coronary events (MI, coronary revascularization, or death from ischemic heart disease) for every Gy of radiation dose to the heart [11]. 

In Hodgkin Lymphoma survivors, the risk of CAD is higher in those with a history of hypertension, at least one CV risk factor (such as diabetes, high cholesterol, or hypertension), or a family history of CAD [10]. Similarly, a study by Darby et al. found that a personal history of CAD, the presence of CV risk factors, and smoking increased the likelihood of major coronary events in breast cancer patients after chest radiation [11]. Both studies failed to show an interaction between risk factors and radiation exposure, indicating that exposure to radiation therapy increases risk of CAD regardless of CV risk factors.

A recent Danish study of 3,151 Non-Hodgkin’s Lymphoma (NHL) survivors found that both mediastinal radiation therapy and traditional CV risk factors independently increased the risk of MI. Compared to patients without CV risk factors or radiation exposure, those with at least one CV risk factor but no radiation exposure had a threefold higher risk of MI, while those with both factors faced a 6.7-fold increased risk [7]. In a cohort of non-small cell lung cancer (NSCLC) patients treated with radiotherapy between 2004 and 2014, presence of pre-existing CAD increased the risk of major adverse cardiac events by 7-fold during a follow-up time of approximately 2 years, providing further evidence that pre-existing risk factors and CAD worsen outcomes in patients receiving radiotherapy [12]. 

There is also evidence that cancer-specific factors such as laterality may affect CAD risk. Darby, et al. showed that the risk of major coronary events was higher in patients with left-sided breast cancer [11]. Similarly, Wennstig et al. demonstrated that among breast cancer patients receiving adjuvant radiotherapy, those with left-sided disease had a significantly higher risk of CAD compared with those treated for right-sided disease. In this large population-based cohort, left-sided radiotherapy was associated with an approximately 20% higher risk of ischemic heart disease (adjusted hazard ratio ~ 1.18), with risk increasing over time following radiation exposure [13]. 

#### Latency and Clinical Presentation

Radiation-associated coronary artery disease (RICAD) is characterized by a variable and often prolonged latency that depends on age at exposure and baseline cardiovascular risk. In survivors of Hodgkin lymphoma treated at a young age, coronary heart disease typically manifests decades after therapy, with a median latency of approximately 19 years between radiation exposure and clinical disease [10]. Similar long latency periods have been observed in breast cancer survivors, particularly following left-sided radiation [11]. In contrast, older patients and those with established cardiovascular risk factors or pre-existing coronary artery disease may develop clinically apparent disease much earlier after chest radiation. This accelerated presentation is especially evident in lung cancer populations, in whom radiation is delivered later in life and often in the context of smoking and baseline atherosclerosis, suggesting that radiation may act as a potent accelerator of existing coronary disease rather than solely inducing de novo atherosclerosis [6, 12]. 

Clinically, RICAD presents across a wide spectrum, ranging from asymptomatic disease to atypical chest pain, stable angina, or myocardial infarction, with silent ischemia and silent myocardial infarction occurring more frequently than in non-irradiated populations [14]. Distinctive anatomic features include a predilection for proximal and ostial coronary involvement, higher overall plaque burden, and increased rates of multivessel disease. Mediastinal radiation is most commonly associated with disease of the left anterior descending and right coronary arteries, whereas left-sided breast radiation predominantly affects the left anterior descending artery [15]. In addition, a high prevalence of coronary microvascular dysfunction, particularly identified on positron emission tomography, has been reported, suggesting that ischemia may occur even in the absence of flow-limiting epicardial stenoses and contributing to diagnostic challenges in this population [16]. 

#### Monitoring and Management

Due to the increased risk of CAD following chest radiation therapy, increased surveillance is recommended. Consensus statements from the European Society of Cardiology (ESC) and the International Cardio-Oncology Society (ICOS) recommend surveillance for CAD every 5 years [17, 18]. A scientific statement by the American Heart Association (AHA) suggests that in childhood lymphoma survivors receiving combined chemotherapy and chest radiation, CAD surveillance should begin 10 years after radiotherapy and can be repeated every 5 years thereafter [19]. Surveillance for CAD can be achieved using stress echocardiogram, coronary artery calcium scoring, CCTA, or myocardial perfusion imaging using positron emission tomography (PET) and single photon emission computed tomography (SPECT). However, for young patients with low CAD risk, stress echocardiogram is preferred as the lifetime cumulative risk from multiple radiation exposure from coronary CT can be a concern. In addition to this, review of available information such as history, physical exam, CAD risk factor screening and management, baseline electrocardiograms (ECG), transthoracic echocardiography (TTE), and review of conventional chest computed tomography (CT) scans for presence of coronary artery calcification is recommended to risk stratify patients or look for signs of CAD [17]. 

Strategies for medical and non-invasive management of CAD following chest radiation therapy follow established guideline-directed therapy, emphasizing aggressive risk factor modification, antiplatelet therapy, high-intensity statins, beta-blockers, and renin–angiotensin system inhibition when indicated, in accordance with AHA/ACC recommendations [20]. Coronary artery bypass graft (CABG) surgery in patients with previous chest radiation may be challenging due to the conduit vessels being stenosed and fibrotic. Patients having radiation-associated damage to the skin and thoracic vessels, and presence of concomitant radiation-induced pulmonary fibrosis and interstitial lung disease can also worsen outcomes and present challenges with cardiopulmonary bypass access for surgery, for example in cases of severe aortic calcification, or “porcelain aorta” [21]. Despite this, data from the national readmission database shows that there is a similar risk of in-hospital mortality and 90-day readmission risk between patients with and without previous radiation undergoing CABG [22]. For patients with previous radiation therapy exposure being managed with percutaneous coronary intervention (PCI), the use of drug eluting stents is associated with better outcomes compared to bare metal stents [23]. The choice of access and post-intervention management is similar to established guidelines for patients without radiation-associated CAD.

### Valvular Heart Disease

#### Epidemiology

Chest radiation therapy has also been associated with an increased risk of VHD [24]. The reported prevalence varies across studies, from approximately 2% to 37%. This variation may be attributed to differences in the methodologies used to assess VHD, including availability of baseline echocardiographic evaluation prior to radiation exposure and color Doppler imaging [25]. Up to 32% of patients may present with asymptomatic valvular damage [26]. 

Studies investigating patients exposed to mediastinal radiation as part of Hodgkin lymphoma treatment demonstrate that the aortic and mitral valves are most commonly affected, with prevalence increasing with time since radiation [27, 28]. More than 20 years after radiation therapy, the prevalence of at least mild aortic regurgitation and mitral regurgitation ranges between 60 and 74% and 40–52%, respectively. At least mild aortic stenosis is also commonly seen, with rates ranging between 16 and 40% 20 years after radiation therapy [27, 28]. These rates are much higher than what is observed in the general population and also Hodgkin lymphoma patients not undergoing radiotherapy. The reason for left-sided valves being more commonly affected is postulated to be higher pressures in the systemic circulation and proximity to the radiation field [29]. 

#### Risk Factors

The primary risk factor for radiation-induced VHD was the amount of radiation administered to the patient. In the French childhood cancer survivor study of 7,492 survivors with a median follow-up of 30.6 years, individuals with VHD who were irradiated had a significantly higher median radiation dose to the heart (28.26 Gy) compared to the overall cohort (1.37 Gy). The risk of VHD increased by 10% for every 1 Gy increase in the median heart dose (MHD), with a significantly higher risk when the MHD exceeded 15 Gy. At age 65, the cumulative incidence was 3.9%, 8.3%, and 24.2% in subgroups that received 5–20 Gy, 20–40 Gy, and ≥ 40 Gy to the heart, respectively [30]. This finding was also supported by a case-control study comparing 89 patients with VHD to 200 control patients. The study showed that the risk of VHD increased more than linearly with the radiation dose. For doses to the affected valve(s) of ≤ 30, 31–35, 36–40, and > 40 Gy, VHD rates increased by factors of 1.4, 3.1, 5.4, and 11.8, respectively (*P* < 0.001). The approximate 30-year cumulative risks for these dose categories were 3.0%, 6.4%, 9.3%, and 12.4%, respectively [26]. 

Concomitant chemotherapy, especially with anthracyclines like doxorubicin, significantly raises the risk of VHD when combined with radiation therapy. Anthracyclines are known to be cardiotoxic, potentially leading to myocardial fibrosis and valvular injury, and the risk is further amplified when used alongside radiation. High-dose anthracyclines (e.g., doxorubicin ≥ 250 mg/m², epirubicin ≥ 600 mg/m²) and high-dose radiation therapy (≥ 30 Gy) to the heart significantly increase the risk of cardiac dysfunction, including valvular damage, with a synergistic effect when used together. Even with lower doses of these treatments, patients with additional cardiovascular risk factors (e.g., smoking, hypertension, diabetes, dyslipidemia, or obesity) still face an elevated risk of cardiac events [31]. 

A newly recognized risk factor for VHD is the use of treatments that affect splenic function (i.e., splenectomy or radiotherapy involving the spleen). A sub-analysis from the Dutch Childhood Cancer Survivor Study found that, after a median follow-up of 22 years, the incidence of VHD by age 40 was significantly higher in cancer survivors who received such treatments (2.7%, 95% CI 0.4%–4.9%) compared to those who did not (0.4%, 95% CI 0.1%–0.7%) (Gray’s test, *p* = 0.003). This effect remained significant even after adjusting for radiotherapy dose to the cardiac region [32]. Other risk factors are the difference between right and left chest radiation and the impact of newer radiation techniques. Only one study has evaluated the prevalence of VHD between 35,000 patients with right- versus left-sided breast cancer who were exposed to radiation. This study found that the incidence of VHD was higher in patients with left-sided breast cancer compared to those with right-sided breast cancer, with an incidence rate ratio of 1.54 [33]. 

#### Monitoring and Management

An expert consensus statement by ICOS recommends a screening TTE to be performed 5 years following completion of radiotherapy, with repeat studies every 5 years for continued surveillance [17]. Other consensus statements by the AHA [19] and American Society of Clinical Oncology (ASCO) [31] have similar recommendations, with earlier and more frequent studies allowed for higher risk patients. Guidance from the Children’s Oncology Group identifies a higher risk population requiring surveillance with a TTE every 2 years, which is those who have received an anthracycline dose between 100 and 250 mg/m^2^ and more than 15 Gy radiation, or those who have received more than 30 Gy radiation regardless of anthracycline exposure [34]. 

Similar to CAD, surgical intervention for patients with VHD and prior chest radiation therapy may be challenging because of the presence of a “hostile chest,” characterized by pericardial and aortic calcification, increased bleeding risk, impaired wound healing, and pulmonary fibrosis. Consequently, traditional surgical risk scores, such as the Society of Thoracic Surgeons–Predicted Risk of Mortality (STS-PROM) and European System for Cardiac Operative Risk Evaluation (EuroSCORE), may underestimate the true risk of cardiac surgery in these patients. The European Society of Cardiology therefore strongly recommends a multidisciplinary Heart Team approach, involving cardiac surgeons, interventional cardiologists, and cardio-oncology specialists, to assess each patient’s individual surgical risk and guide treatment decisions [21, 35]. 

Based on these discussions, catheter-based percutaneous interventions may be preferred in some patients. Among the different catheter-based valvular interventions, transcatheter aortic valve replacement (TAVR) is the most studied. A recent meta-analysis compared TAVR outcomes between patients with and without a history of chest radiation therapy. They found that the radiation group had more than 5-fold higher odds of reintervention and two-fold higher odds of HF exacerbation in the peri-procedural period. Other outcomes such as short and mid-term survival, procedural success and complications, need for pacemaker, and perioperative MI and strokes were similar between groups [36]. Surgical aortic valve replacement (SAVR) outcomes are significantly worse in patients with a history of chest radiation therapy, with a previous matched cohort study reporting an 8-fold higher risk of long-term mortality compared to patients without a history of radiation therapy [37]. Transcatheter and surgical interventions involving other valves in patients with prior chest radiation therapy is less studied.

### Cardiomyopathy

#### Epidemiology

After chest radiation therapy, patients are at an increased risk of developing cardiomyopathy either due to direct damage to the cardiomyocytes or due to damage to other structures of the heart. The prevalence of diastolic dysfunction after chest radiation ranges between 14% − 29% in different cohorts [38, 39], and it appears to be more common than overt systolic dysfunction, which is consistent with the pathophysiology of increased fibrosis and reduced compliance. A dose-dependent relationship between radiation dose and risk of heart failure with preserved ejection fraction (HFpEF) has been demonstrated, but not for heart failure with reduced ejection fraction (HFrEF) [40]. However, more recent studies have shown that global longitudinal strain (GLS) becomes increasingly impaired with time in women treated with chest radiation [41], indicating some degree of subclinical systolic dysfunction.

#### Risk Factors

The risk of cardiomyopathy following chest radiation is amplified by several patient- and treatment-related factors, including underlying cardiovascular comorbidities and concurrent exposure to cardiotoxic systemic therapy. In a cohort of mediastinal irradiation survivors, diastolic dysfunction was identified in ~ 14% of patients (mild 9%, moderate 5%), and was associated with worse event-free survival compared with patients with normal diastolic function (HR 1.66, 95% CI 1.06–2.40) [39]. Increasing age and metabolic risk also appear to play a role. In adult childhood cancer survivors, echocardiographic abnormalities were more frequently observed among patients with cardiometabolic risk profiles, including those meeting criteria for metabolic syndrome, reinforcing that traditional cardiovascular risk factors may compound treatment-related myocardial vulnerability [42]. Additional comorbid disease burden further increases risk: exercise-induced ischemia was more frequent among irradiated patients with diastolic dysfunction (23% vs. 11%, *p* = 0.008), consistent with interaction between radiation injury and coronary disease physiology [39]. Finally, radiation-related risk appears to be potentiated by systemic therapy, as long-term Hodgkin lymphoma survivor data demonstrate that heart failure risk rises with cardiac radiation exposure and is further increased by anthracycline exposure, supporting an additive (or synergistic) cardiotoxic effect when both modalities are present [40, 43]. 

#### Monitoring and Management

A position statement from ICOS mentions that obtaining a baseline ECG and TTE in patients receiving chest radiation therapy can be useful. Furthermore, surveillance imaging after completion of therapy (TTE and/or cardiac MRI) is recommended, with timing guided by baseline risk; in high-risk patients, the first surveillance study may be obtained as early as 6–12 months after therapy, with repeat TTEs every 5 years for long-term monitoring [17]. Management of cardiomyopathy and heart failure in patients with previous radiation therapy aligns with standard guidelines [44]. In terms of advanced heart failure therapy, a recent analysis of heart transplant outcomes from the United Network of Organ Sharing (UNOS) registry showed that patients with chemotherapy or radiation related cardiomyopathy had longer waiting times (mean 223 days) and worse survival [45]. Older UNOS-era data further support this risk signal in radiation-associated restrictive phenotypes, with the XRT subgroup demonstrating lower survival after transplant (1-, 5-, and 10-year survival: 71%, 47%, and 32%) and higher mortality versus other restrictive cardiomyopathy etiologies (unadjusted HR 1.81, *p* = 0.002) [46]. 

Durable mechanical circulatory support can also be considered in carefully selected patients with cancer therapy-related cardiomyopathy. In the contemporary Interagency Registry for Mechanically Assisted Circulatory Support (INTERMACS) era (2008–2017), patients with anthracycline-induced cardiomyopathy supported with continuous-flow left ventricular assist devices (LVADs) had similar 12-month survival compared with idiopathic dilated and ischemic cardiomyopathy (85.1% vs. 86.0% vs. 80.2%), with no significant difference in adjusted mortality (anthracycline vs. idiopathic: aHR 1.12, 95% CI 0.88–1.43; anthracycline vs. ischemic: aHR 0.98, 95% CI 0.76–1.28) [47]. In a separate INTERMACS analysis of patients with a history of cancer undergoing durable LVAD implantation, cancer history was associated with mildly reduced survival (median 3.72 vs. 3.97 years; HR 1.14, 95% CI 1.04–1.26; *p* = 0.005) and lower subsequent transplant rates, but importantly mortality was not higher in the subgroup with cancer therapy–related cardiomyopathy, supporting LVAD as a feasible bridge-to-transplant or destination strategy when oncologic prognosis and bleeding risk are acceptable [48]. Data specifically isolating radiation-induced cardiomyopathy among LVAD recipients remain limited, and decisions should be individualized within a multidisciplinary cardio-oncology and advanced heart failure team.

### Pericardial Disease

#### Epidemiology

Pericardial effusion after chest radiation can occur weeks, months, or years after treatment and is caused by radiation-induced inflammation and scarring of the pericardium. The percentage of patients who develop pericardial effusions after chest radiation for cancer varies, with some studies showing a range of approximately 24% to 52% for specific cancers like esophageal cancer. Other studies report lower overall incidences, such as 2% to 6% at doses below 40 Gy, while rates can increase with higher doses and larger radiation fields [49, 50]. The degree of pericardial involvement is variable, ranging from asymptomatic to clinically significant disease requiring pericardiocentesis, and in some cases, causing tamponade [50]. Chronic pericarditis may develop post-radiation with previously reported incidences of 7–20% [51], although modern radiation techniques have made them less common [19]. Radiation can also result in pericardial thickening and calcification with resultant constrictive pericarditis [17]. 

#### Risk Factors

Several factors increase the risk of pericardial disease in patient receiving radiotherapy. In two studies, radiation dose emerged as an important risk factor for subsequent development of pericardial effusion. In these studies, left tumor location, use of adjuvant chemotherapy, cardiac comorbidities, and stage of cancer emerged as additional independent risk factors [49, 50]. 

#### Monitoring and Management

In patients with radiation exposure to the heart, an expert statement from ICOS recommends evaluation of the pericardium with TTE starting 5 years after completion of therapy, with repeat studies every 5 years for continued surveillance. In patients with suspected constrictive pericarditis, multimodal imaging techniques including cardiac CT and cardiac MRI may be necessary [17]. In cases where the diagnosis of constrictive pericarditis with noninvasive methods are nondiagnostic or equivocal, invasive assessment with right heart catheterization may be necessary.

Management of pericardial sequelae of radiation therapy should follow standard clinical practices as employed for other etiologies, including pericardiocentesis and pericardiectomies where indicated. According to a recent meta-analysis, the risk of mortality after pericardiectomy for radiation induced constrictive pericarditis is more than three-fold higher compared to idiopathic constrictive pericarditis [52]. In a cohort of patients undergoing pericardiectomy for mediastinal radiation induced constrictive pericarditis, the 5- and 10- year survivals were 53.4% and 32.1% respectively, with older age emerging as a risk factor for mortality [53]. 

### Arrhythmias

#### Epidemiology

The prevalence of arrhythmias in patients exposed to radiotherapy varies depending on the type of cancer and the site of radiation. A study by Wang et al. investigated the incidence of arrhythmias in 112 patients who underwent radiotherapy as part of lung cancer treatment. After a median follow-up of 23 months, new-onset arrhythmias were observed in 10.7% of patients, with atrial fibrillation being the most common (66.7%), followed by atrial flutter (16.7%) [54]. Similarly, Atkins, et al. found that 17.1% of their lung cancer patients undergoing radiation experienced significant arrhythmias, with nearly half of them being atrial fibrillation and about 15% being atrial flutter [55]. In another cohort of 746 breast cancer patients, those who received chest radiation therapy had a 4.8% cumulative 10-year risk of developing significant arrhythmias [56]. Another study examining 238 patients with esophageal cancer who received radiotherapy reported an atrial fibrillation incidence of 21.4%, with a cumulative one-year incidence of 19.5% [57]. 

#### Risk Factors

Previous studies have suggested a potential association between radiation dose and the risk of arrhythmias. A case-control study of patients with breast cancer treated with radiotherapy observed a non-significant trend toward a higher risk of arrhythmia with increasing radiation dose to the right atrium [58]. In contrast, a cohort study of 238 patients with esophageal cancer found that higher radiation exposure to the left atrium was independently associated with the development of atrial fibrillation, and patients with preexisting atrial fibrillation experienced worse disease progression. Other risk factors for atrial fibrillation were a history of surgical resection and increasing age [57]. 

#### Monitoring and Management

It is recommended that a standard ECG should be obtained at each cardiovascular screening visit following chest radiation therapy, which can occur every 5 years or earlier/ more frequently depending on risk factors and presence of symptoms [19]. Besides this, investigations, interventions, and other management strategies align with standard guidelines [21, 23]. 

There are special considerations for patients with cardiac implantable electronic devices (CIEDs), including pacemakers and implantable cardioverter-defibrillators (ICDs), who are undergoing radiotherapy. Careful planning before radiotherapy is essential as ionizing radiation may cause device malfunction. Ideally, planning and management should involve a multidisciplinary team comprising of a radiation oncologist, medical physicist, a cardio-oncologist, and an electrophysiologist experienced in CIED management when available. Treatment planning should minimize radiation exposure to the CIED whenever feasible. Baseline assessment should include the device type, recent interrogation, pacemaker dependence, and lower pacing rate. Patients should be risk-stratified by the multidisciplinary team prior to radiotherapy, with high-risk patients characterized by ICD presence, pacemaker dependence, anticipated device dose > 5 Gy, and exposure to neutron-producing radiation (e.g., photon energies > 10 MV, electron energies > 20 MeV, or proton therapy). High-risk patients require continuous cardiac monitoring during treatment, access to external pacing/defibrillation, temporary asynchronous pacing in pacemaker-dependent patients, and deactivation of ICD therapies. Device relocation may be considered selectively based on individualized assessment and shared decision-making between the patient and the multidisciplinary team. Device interrogation should be performed before and after RT, with follow-up evaluation after completion of therapy and more frequent monitoring in higher-risk patients [59]. 

## Measures to Mitigate Radiation Associated Cardiotoxicity

### Radiation Therapy Techniques

As described above, radiation associated cardiotoxicity follows a dose-dependent relationship. Hence, the ASCO and ICOS recommend minimizing the dose of radiation to the heart wherever possible. This can be achieved by using the minimal dose of radiation while maintaining clinical efficacy and using specialized techniques such as intensity modulated radiation therapy (IMRT), deep inspiratory breath holding, and involved node radiotherapy [17, 31]. 

IMRT is a form of 3-dimensional conformal radiotherapy (3DCRT) that uses computed tomography imaging to map the target and surrounding tissues. It utilizes ‘inverse algorithms’ to plan and modify radiation delivery characteristics to maximize the dose to the target and minimize the dose to nearby normal tissue [19]. Compared to standard 3DCRT, IMRT effectively reduces the mean heart dose of radiation [60]. Moreover, the long-term risk of major adverse cardiac events was reduced by 60% with IMRT compared to standard 3DCRT in a previous study on NSCLC patients [12]. 

Deep inspiratory breath holding during radiotherapy flattens the diaphragm and expands the lungs, increasing the distance between the heart and the chest wall. Previous studies have shown that this technique approximately halves the mean heart dose and mean left anterior descending (LAD) coronary artery dose of radiation exposure [61]. 

Prone positioning has been investigated as an alternative to supine positioning to reduce radiation exposure to non-target tissues during breast cancer radiotherapy. Although prone positioning consistently reduces ipsilateral lung dose, its effect on cardiac radiation exposure is less consistent and appears to vary by breast laterality and breathing technique. In the large multicenter REQUITE study, prone positioning was associated with a lower mean heart dose in patients with left-sided breast cancer but a higher mean heart dose in those with right-sided disease [62]. This was corroborated by Lohm, et al., who also found a higher mean heart dose in right-sided breast cancer patients undergoing radiotherapy in prone versus supine position [63]. A meta-analysis of 751 patients similarly found that prone positioning reduced mean heart dose and left anterior descending coronary artery dose compared with supine positioning when both were performed with free breathing [64]. However, comparative dosimetric studies have demonstrated that supine positioning with deep inspiration breath-hold achieves lower cardiac doses than prone positioning with free breathing [65], highlighting the importance of breathing technique in minimizing cardiac exposure. Conversely, when prone and supine positioning are both performed with deep inspiration breath-hold, prone positioning may provide a lower mean heart dose than supine positioning [64]. Thus, the optimal positioning strategy should be individualized according to breast laterality and the breathing technique used, with the goal of minimizing cardiac as well as pulmonary radiation exposure.

### Cardiovascular Risk Factors

The presence of cardiovascular risk factors and comorbid cardiovascular disease significantly increases the risk of radiation associated cardiotoxicity. For example, presence of at least one cardiovascular risk factor increased the 10-year estimated risk of MI from 3.9% to 10.2% in lymphoma patients receiving chest radiation therapy [7]. Similarly, presence of metabolic syndrome increases the risk of diastolic dysfunction, and smoking enhances the risk of major cardiac events following chest radiation therapy [42, 66]. As such, preventive measures for regular screening and strict optimization of cardiovascular risk factors should be employed in patients undergoing or with a history of chest radiation therapy.

### Lifestyle Modification

Lifestyle optimization represents an important strategy to mitigate radiation-associated cardiotoxicity, particularly because radiation exposure often accelerates atherosclerosis and interacts with traditional cardiovascular risk factors. Consistent with the 2019 ACC/AHA Guideline on the Primary Prevention of Cardiovascular Disease, comprehensive lifestyle interventions: including a heart-healthy diet, routine physical activity, weight management, and smoking cessation; form the foundation of long-term cardiovascular risk reduction and are recommended across cardiovascular risk strata [67]. In the setting of chest radiation, these interventions are especially relevant because metabolic and inflammatory pathways contribute to endothelial dysfunction, myocardial fibrosis, and progression to clinical coronary disease or heart failure. Exercise may offer additional mechanistic protection beyond risk-factor control: a systematic review evaluating exercise as a countermeasure to radiation toxicity reported that compared with sedentary or usual-care controls, exercise reduced radiation-induced oxidative stress, inflammation, and DNA damage, with evidence of benefit across cellular, functional, and clinical outcomes despite heterogeneity in study designs and exercise protocols [68]. Similarly, in patients at risk for radiation-induced coronary heart disease, expert review data emphasize that aggressive management of modifiable cardiovascular risk factors; including lipid control, blood pressure optimization, diabetes prevention, and smoking cessation; remains central to prevention and may reduce the likelihood of radiation-accelerated coronary disease and downstream heart failure. A previous study found a dose-dependent reduction in the risk of cardiovascular events with vigorous exercise, with a 51% reduction in risk at an exercise intensity of ≥ 9 MET-hours/week [69]. 

### Pharmacotherapy

Data for pharmacotherapy for mitigation of radiation associated cardiotoxicity in humans is limited. Animal studies suggest that statins, aspirin, angiotensin converting enzyme inhibitors, and dexrazoxane may be promising in preventing radiation associated cardiotoxicity, likely by ameliorating oxidative stress and inflammation [70–72]. Recent studies have shown benefits of using sodium glucose cotransporter-2 inhibitors in cancer patients receiving cardiotoxic chemotherapy [73], but data for patients receiving radiation therapy is limited. Future studies must ascertain whether use of these drugs can reduce the risk of radiation associated cardiotoxicity in humans. High quality evidence from randomized controlled trials is needed to demonstrate the efficacy of pharmacological prevention of RIHD.

## Conclusion

Chest radiation therapy plays a critical role in the treatment of many cancers, but appears to increase the risk of cardiotoxicity in a dose-dependent manner. A wide range of cardiovascular complications following chest radiation therapy has been described, including coronary artery disease, valvular heart disease (most commonly involving the aortic valve), cardiomyopathy, pericardial disease, and arrhythmias. Besides radiation dose, co-existence of other cardiovascular risk factors and cardiovascular comorbidities amplifies the risk of cardiotoxicity. As such, modern radiotherapy techniques must be employed to minimize the radiation dose to the heart, and preventive strategies should be implemented for aggressive control of other modifiable cardiovascular risk factors. Cancer survivors with a history of chest radiation therapy benefit from routine surveillance of cardiovascular diseases. Future studies are warranted to investigate whether pharmacological strategies can mitigate the risk of chest radiation therapy associated cardiotoxicity and cost-effectiveness of different surveillance strategies based on individualized risk assessment.

## Key References

- Bates JE, Rancati T, Keshavarz H, Gagliardi G, Aznar MC, Howell RM, et al. Cardiac Disease in Childhood Cancer Survivors Treated With Radiation Therapy: A PENTEC Comprehensive Review. Int J Radiat Oncol Biol Phys. 2024;119:522–32.
  - ○ This recent comprehensive review demonstrates the dose-dependent nature of chest radiation induced heart disease.
- Miller ED, Wu T, McKinley G, Slivnick J, Guha A, Mo X, et al. Incident Atrial Fibrillation and Survival Outcomes in Esophageal Cancer following Radiotherapy. Int J Radiat Oncol Biol Phys. 2024;118:124–36.
  - ○ Key study demonstrating the high burden of atrial fibrillation following chest radiation for esophageal cancer.
- Mitchell JD, Cehic DA, Morgia M, Bergom C, Toohey J, Guerrero PA, et al. Cardiovascular Manifestations From Therapeutic Radiation: A Multidisciplinary Expert Consensus Statement From the International Cardio-Oncology Society. JACC CardioOncol. 2021;3:360–80.
  - ○ Current society expert consensus recommendations for the surveillance of radiation-induced heart disease and strategies for risk mitigation.

## Data Availability

No datasets were generated or analysed during the current study.

## Abbreviations

AHA: American Heart Association
ASCO: American Society of Clinical Oncology
ATM: Ataxia telangiectasia–mutated
CABG: Coronary artery bypass graft
CAD: Coronary artery disease
CAC: Coronary artery calcium
CI: Confidence interval
CIED: Cardiac implantable electronic device
CT: Computed tomography
CCTA: Coronary computed tomographic angiography
CV: Cardiovascular
DNA: Deoxyribonucleic acid
ECG: Electrocardiogram
ESC: European Society of Cardiology
EuroSCORE: European System for Cardiac Operative Risk Evaluation
GLS: Global longitudinal strain
Gy: Gray
HF: Heart failure
HFpEF: Heart failure with preserved ejection fraction
HFrEF: Heart failure with reduced ejection fraction
HR: Hazard ratio
ICOS: International Cardio-Oncology Society
ICD: Implantable cardioverter-defibrillators
IMRT: Intensity-modulated radiation therapy
INTERMACS: Interagency Registry for Mechanically Assisted Circulatory Support
LAD: Left anterior descending (coronary artery)
LVAD: Left ventricular assist device
MHD: Mean heart dose
MI: Myocardial infarction
NSCLC: Non–small-cell lung cancer
NHL: Non-Hodgkin lymphoma
OR: Odds ratio
PCI: Percutaneous coronary intervention
PET: Positron emission tomography
RIHD: Radiation-induced heart disease
RICAD: Radiation-associated coronary artery disease
ROS: Reactive oxygen species
SAVR: Surgical aortic valve replacement
SPECT: Single photon emission computed tomography
STS-PROM: Society of Thoracic Surgeons–Predicted Risk of Mortality
TAVR: Transcatheter aortic valve replacement
TGF-β: Transforming growth factor beta
TTE: Transthoracic echocardiography
UNOS: United Network of Organ Sharing
VHD: Valvular heart disease
XRT: Radiotherapy (radiation therapy)
